# Effect of deflazacort on pregnancy outcome in kidney transplant patients: should we change the immunosuppressant before conception?

**DOI:** 10.1186/s12882-019-1346-6

**Published:** 2019-05-14

**Authors:** Bo Hyon Yun, Dong Jin Joo, Seok Kyo Seo, Si Hyun Cho, Young Sik Choi, Byung Seok Lee

**Affiliations:** 1 Department of Obstetrics and Gynecology, Severance Hospital, Yonsei University College of Medicine, 50 Yonsei-ro, Seodaemun-gu, Seoul, 03722 Republic of Korea; 2 Institute of Women’s Life Medical Science, Yonsei University College of Medicine, Seoul, Republic of Korea; 3 Department of Transplant Surgery, Severance Hospital, Yonsei University College of Medicine, Seoul, Republic of Korea; 4 Department of Obstetrics and Gynecology, Gangnam Severance Hospital, Yonsei University College of Medicine, Seoul, Republic of Korea

**Keywords:** Kidney transplant, Immunosuppressant, Pregnancy

## Abstract

**Background:**

Despite the good prognosis in patients with transplant organs, limited evidence is available on how immunosuppressants affect pregnancy. The aim of this study was to determine whether immunosuppressant use affects the pregnancy outcome and to identify whether there is any need to change the immunosuppressant before the patient tries to conceive.

**Methods:**

This retrospective cohort study included women with previous kidney transplantation history who visited the Department of Obstetrics and Gynecology for either infertility or antenatal care between January 2005 and May 2016. A total of 40 cases (36 women) met the inclusion criteria. Statistical analyses were performed using SAS version 9.4.

**Results:**

There were no differences in the immunosuppressant regimen between the pregnant and non-pregnant groups (never-pregnant+miscarriage) (*P* = 0.73). Individual immunosuppressant use was significantly different in terms of pregnancy outcome among the never-pregnant, miscarriage, and clinical pregnancy groups (azathioprine, *P* = 0.01; deflazacort, *P* < 0.0001). Only deflazacort use differed significantly between the clinical pregnancy and non-pregnant groups (*P* = 0.003). After adjusting for factors that may affect pregnancy outcome, deflazacort use remained significantly associated with a decreased odds ratio for clinical pregnancy (*P* = 0.02). Cox regression analysis also showed that deflazacort use was the only remaining factor that could hinder the success of clinical pregnancy (*P* = 0.03).

**Conclusions:**

Our study suggests that the type of immunosuppressive regimen may not affect the success of clinical pregnancy. However, deflazacort may decrease the possibility of clinical pregnancy in women with kidney transplant when they try to conceive.

## Introduction

Since the first successful kidney transplantation performed in 1954 by Dr. Joseph E. Murray, the number of people who live longer and healthier lives continues to increase each year. About 126,670 patients underwent organ transplantation in 2015 worldwide, with the kidney being the most commonly transplanted organ (84,347 cases), according to statistics from the Global Observatory on Donation and Transplantation (www.transplant-observatory.org). The first successful pregnancy after kidney transplantation was reported in 1956 [1] and the first pregnancy following in vitro fertilization-embryo transfer (IVF-ET) in a kidney transplant patient was in 1995 [2]. Extensive discussion has been conducted regarding the adequate conditions necessary before trying to conceive, such as stable blood pressure (BP), a normally functioning transplant organ, and infrequent graft rejections [3, 4]. For years, observations suggested that transplantation and immunosuppressant use may not interfere with the fertility of women [5], but may affect the pregnancy outcome such as intrauterine growth restriction and preeclampsia, leading to preterm delivery [6]. Given the effect of chronic disease and renal dysfunction, which may affect steroid hormone metabolism, infertility related to anovulation has been suggested as a possible infertility factor; however, after transplantation, the increase in ovulation resumption may lead to incidental pregnancy [5]. The number of patients trying to conceive is increasing, owing to improved prognosis and well-being after transplantation.

However, there is a paucity of information on whether immunosuppressant use affects the achievement of pregnancy or the pregnancy outcome. When a kidney transplant patient considers pregnancy, certain immunosuppressants, such as mycophenolate mofetil, may be substituted because of their potential risk for fetal congenital orofacial anomalies and miscarriage; however, even this remains unclear as no distinct pattern of congenital anomalies has been identified as of yet [7]. The aim of this study was to determine whether immunosuppressant use affects the pregnancy outcomes of women with kidney transplants and to identify whether there is any need to change the immunosuppressant before the patient tries to conceive.

## Methods

### Study population

**Fig. 1:**
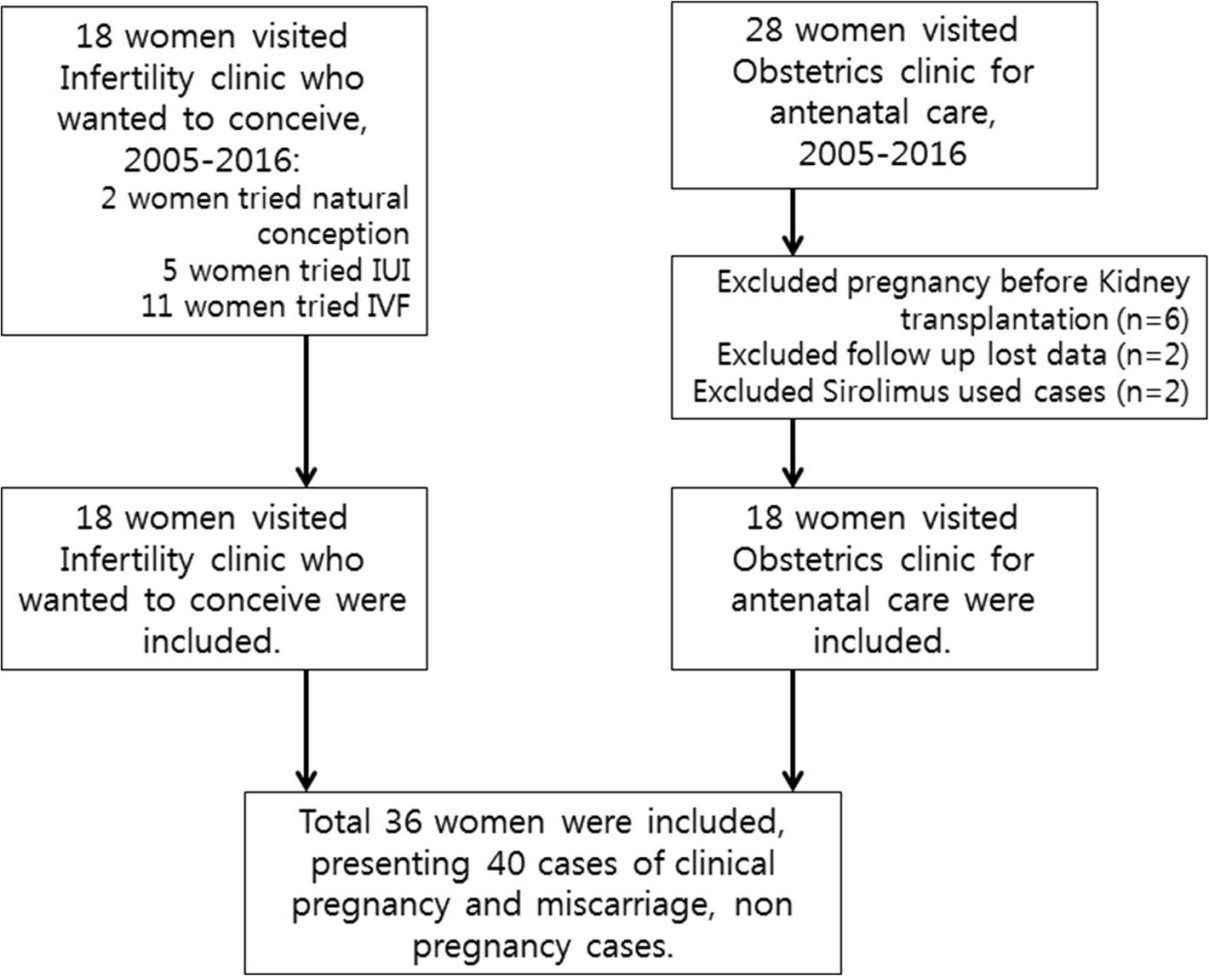
Flow chart of participant inclusion in the study

Women with a history of kidney transplantation who visited the Department of Obstetrics and Gynecology for infertility or antenatal care from January 2005 to May 2016 in Severance Hospital, Yonsei University College of Medicine were included. Women who were pregnant before transplantation (*n* = 6), cases lost to follow-up (*n* = 2), and those who used certain immunosuppressants, such as sirolimus (*n* = 2) were excluded (Fig. 1). Finally, 40 cases (36 women) were included in this study: 18 cases of 18 infertile women who visited the infertility clinic (infertile group) and 22 cases of 18 pregnant women who visited the antenatal clinic (fertile group) after achieving successful conception within a year of trying to conceive. Prednisolone (Pred) was used as a first line steroid. Deflazacort (DFZ) was chosen instead of Pred in patients with diabetes, osteoporosis, and Cushing syndrome caused by prolonged use of corticosteroids.

Primary infertility was diagnosed in those who had tried to conceive for more than 1 year without contraception, and secondary infertility was diagnosed in those who had a previous history of pregnancy but failed to conceive for more than 1 year. The duration of attempted conception, cause of end-stage renal disease (ESRD), time since the transplantation, and whether the patient had graft rejection and hypertension were assessed through a retrospective chart review. The time of data collection for each factor, including the type of immunosuppressant medication, was within the month of the last menstruation in the clinical pregnancy and miscarriage groups or at the start of attempting conception in the never-pregnant group. The never-pregnant and miscarriage groups were merged into the non-pregnant group in analyses to compare with the clinical pregnancy group [8].

### Measurements

Patients’ BP was measured using an automated oscillometric measurement while seated with their arm at heart level. Hypertension was diagnosed if systolic BP ≥140 mmHg, diastolic BP ≥90 mmHg, or antihypertensive medication was currently used. Blood samples were taken from the antecubital vein after at least 8 h of fasting. Blood tests commonly included blood urea nitrogen (BUN) and creatinine (Cr). Some patients who visited the infertility clinic underwent laboratory testing for hormones, including the follicle-stimulating hormone, luteinizing hormone, estradiol, and anti-Müllerian hormone. Renal function was assessed by estimated glomerular filtration rate (eGFR) [9, 10], using the following formula: Modification of Diet in Renal Disease (MDRD) 175 × Cr-1.154 × age-0.203 × 0.742.

To confirm the pregnancy, transvaginal ultrasonography was performed by experienced gynecologists using the available ultrasound systems (Accuvix V20 Prestige, Medison Co., Seoul, Korea; iU22, Philips Healthcare, WA, USA; and Voluson E8, GE Medical Systems, Zipf, Austria). Clinical pregnancy was defined as the presence of an intrauterine gestational sac with a pulsating fetal heartbeat detected by ultrasonography. Miscarriage was defined as confirmed intrauterine gestational sac without fetal heartbeat on ultrasonography.

All women with infertility underwent testing for tubal patency by hysterosalpingography and evaluation of pelvic anatomy by ultrasonography. Semen parameters were interpreted using the World Health Organization (2010) criteria [11]. The causes of infertility were unexplained infertility, male factor, tubal factor, decreased ovarian reserve, and endometriosis. The patients who visited the infertility clinic decided to proceed with intrauterine insemination (IUI; *n* = 5) or IVF-ET (*n* = 11) based on the cause of their infertility. Women with unexplained infertility in conjunction to irregular menstruation tried to conceive naturally using ovulation induction (*n* = 2).

### Statistical analysis

Data was analyzed using the SAS version 9.4 (SAS Institute, Cary, NC, USA). The Student t-test and one-way analysis of variance were used to compare the means for normally distributed continuous variables. The Mann-Whitney U-test and Kruskal-Wallis test were used to analyze non-parametrically distributed variables. The Fisher exact and chi-square tests were used to assess categorical variables. Univariate and multivariate logistic regression analyses were also performed. The logistic model included the factors that showed significance on the univariate analysis. Given the uneven distribution of the adjusting factors, the Firth-type bias-reduced logistic regression was used for adjustment. Multicollinearity, goodness of fit, and the predictive power of the logistic model were checked. Based on the logistic model, Cox’s regression analyses for clinical pregnancy and trial period of pregnancy were performed. A *P*-value < 0.05 was considered statistically significant.

## Results

A total of 40 cases in 36 kidney transplant women were included in this study: 10 cases who had never been pregnant, 9 cases with miscarriage, and 21 cases with clinical pregnancy. One patient in the fertile group had three miscarriages. Two other patients in the fertile group each had one delivery and one miscarriage. Among the infertile women, 12 and 6 women had primary and secondary infertility, respectively. Of these women, 11 had an unexplained cause for infertility, 2 had infertility due to male factor, 4 had decreased ovarian reserve, and 1 had severe endometriosis (retaining duplicate causes). In the infertile group, 2, 4, and 12 women attempted pregnancy through natural conception, IUI, and IVF-ET, respectively. All women in the fertile group conceived naturally. Twenty-one cases of clinical pregnancy were achieved through natural conception in 15 women, IUI in 2 women, and IVF-ET in 2 women. The mean age of all participants was 33.8 ± 3.4 years. Mean duration of attempted conception was 17.6 ± 18.2 months and average time interval from the time of kidney transplant to the time they tried conceiving was 5.9 ± 4.1 years.

Table 1 shows the patients’ baseline characteristics categorized by pregnancy outcome. The median age and BP were the lowest in the clinical pregnancy group, without a significant difference. Serum BUN level (17.2 mg/dL, *P* = 0.04) was low and eGFR (60.72, *P* = 0.02) was significantly high in the clinical pregnancy group compared to other groups. The proportion of patients with diabetes mellitus was significantly different among the groups, with two cases occuring in the never-pregnant group (*P* = 0.008). The graft rejection rate was significantly different among the three groups; the incidence of rejection was highest in the miscarriage group and lowest in the clinical pregnancy group (never-pregnant 30%, miscarriage 66.7%, clinical pregnancy 9.5%, *P* = 0.01). The cause of ESRD was predominantly nephrotic syndrome in the clinical pregnancy group (glomerulonephritis 42.9%, immunoglobulin A nephropathy 33.3%) and never-pregnant group (glomerulonephritis 40%, immunoglobulin A nephropathy 20%), while systemic lupus erythematous (SLE) (33.3%) was highest in the miscarriage group, but without significant difference among the groups (*P* = 0.13). Immunosuppressant use was analyzed in terms of the regimen and type of medication. The immunosuppressants analyzed were as follows: cyclosporine (CyA), tacrolimus (TAC), DFZ, Pred, azathioprine (AZT), and mycophenolate mofetil (MMF). The regimens were grouped as follows: CI + ST + AP, CI + ST, and CI + AP (CI, calcineurin inhibitor: CyA and TAC; ST, steroid: DFZ and Pred; AP, antiproliferative drug: AZT and MMF). Among the never-pregnant, miscarriage, and clinical pregnancy groups, the use of AZT (*P* = 0.01) and DFZ (*P* < 0.0001) was significantly different; however, there was no difference in the immunosuppressive regimen. None of the patients consumed DFZ on account of planning for pregnancy. All patients started DFZ directly following kidney transplantation as a substitute for Pred according to medical indications. There were only two patients with diabetes among the 36 women in our study.

**Table 1:** Baseline characteristics of participants according to the pregnancy outcome

	Never-pregnant (*n* = 10)	Miscarriage (*n* = 9)	Clinical pregnancy(*n* = 21)	*P* value
Age (years)	34.5 (29–42)	36 (30–39)	33 (27–41)	0.44
Duration tried to concieve (months)	44 (16–108)	48 (6–96)	22 (6–96)	0.1
Years since transplantation (years)	4 (2–8)^a^	7 (2–18)^a^	5.5 (1–14)	0.08
SBP (mmHg)	123 (113–144)	135 (100–162)	120 (100–145)	0.44
DBP (mmHg)	80 (70–92)	76 (60–96)	72 (58–96)	0.2
Serum BUN (mg/dL)	18.85 (8.4–34.4)	25.4 (18.7–37.2)^b^	17.2 (10.2–35.3)^b^	0.04
eGFR	55.08 (22–96.6)	39 (9.0–76)^b^	60.72 (37–89.7)^b^	0.02
Hypertension				0.59
No	4 (40%)	2 (22.2%)	5 (23.8%)	
Yes	6 (60%)	7 (77.8%)	16 (76.2%)	
Diabetes melitus				0.04
No	8 (80%)	9 (100%)	21 (100%)	
Yes	2 (20%)	0	0	
Graft rejection				0.01
No	7 (70%)	3 (33.32%)	19 (90.5%)	
Yes	3 (30%)	6 (66.7%)^b^	2 (9.5%)^b^	
Cause of ESRD				0.13
Unknown	1 (10%)	2 (22.2%)	5 (25%)	
SLE	1 (10%)	3 (33.3%)	0	
Glomerulonephritis	4 (40%)	2 (22.2%)	9 (42.9%)	
Ig A nephropathy	2 (20%)	1 (11.1%)	7 (33.3%)	
Congenital anomaly	1 (10%)	1 (11.1%)	0	
Diabetes melitus	1 (10%)	0	0	
Immunosuppressant regimen				0.3
CI	1 (10%)	2 (22.2%)	6 (28.6%)	
CI + ST + AP	6 (60%)	1 (11.1%)	5 (23.8%)	
CI + ST	3 (30%)	5 (55.6%)	9 (42.9%)	
CI + AP	0	1 (11.1%)	1 (4.8%)	
Immunosuppressant use				
CyA	3 (30%)	2 (22.2%)	9 (45%)	0.52
TAC	6 (60%)	7 (77.8%)	12 (57.1%)	0.55
DFZ	9 (90%)	1 (11.1%)	2 (9.5%)	< 0.0001
Pred	2 (20%)	6 (66.7%)	13 (61.9%)	0.06
Aza	5 (50%)	0	2 (9.5%)	0.01
MMF	2 (20%)	2 (22.2%)	4 (19%)	0.98

Data are presented as the median (minimum to maximum). The *P*-values were obtained using the analysis of variance and the Kruskal-Wallis test for continuous variables, and the chi-square test was used for analyzing categorical variables. Post hoc analysis using the Tukey method was performed, which showed a significant difference between the non-pregnant group and the miscarriage group^a,^ and the miscarriage group and clinical pregnancy group^b^

*Note:* Duration, period of attempted conception; SBP, systolic blood pressure; DBP, diastolic blood pressure; BUN, blood urea nitrogen; eGFR, estimated glomerular filtration rate; ESRD, end stage renal disease; SLE, systemic lupus erythematous; Ig, immunoglobulin; CI, calcineurin inhibitor; ST, steroid; AP; antiproliferative drug; CyA, cyclosporine; TAC, tacrolimus; DFZ, deflazacort; Pred, predinosolone; Aza, azathioprine; MMF, mycophenolate mofetil

Table 2 presents a comparison of immunosuppressant use among never-pregnant, miscarriage, and clinical pregnancy groups. The clinical pregnancy group had no significant difference in the regimen used. Regarding each medication separately, DFZ use was significantly different between the non-pregnant and clinical pregnancy groups (52.6% vs. 9.5%, *P* = 0.003). Use of other immunosuppressants was similar between these two groups. We tried to determine whether there would be any difference if we grouped the patients according to the presence of a gestational sac on the ultrasonogram to either the implantation or the non-implantation group for failed pregnancies (data not shown). Steroid use remained significantly different between the two groups: DFZ use in non-implantation vs. implantation groups (88.9% vs. 12.9%, *P* < 0.0001); and Pred use in non-implantation vs. implantation groups (22.2% vs. 61.3%, *P* = 0.04). Additionally, AZT use was significantly different between the non-implantation and implantation groups (55.6% vs. 6.5%, *P* = 0.001). The immunosuppressive regimen was not different between the implantation and non-implantation groups. There were no significant differences in the method of conception between the non-pregnant and clinical pregnancy groups according to the immunosuppressant used, except for DFZ (*P* = 0.02, Table 3) and MMF (*P* = 0.02, Table 3).

**Table 2:** Differences in immunosuppressant use and the pregnancy outcome

		Non-pregnant (*n* = 19)	Clinical pregnancy (*n* = 21)	*P* value
				0.73
Regimen	CI	3 (15.8%)	6 (28.6%)	
	CI + ST + AP	7 (36.8%)	5 (23.8%)	
	CI + ST	8 (42.1%)	9 (42.9%)	
	CI + AP	1 (5.3%)	1 (4.8%)	
Immunosuppressant use	CyA	5 (26.3%)	9 (42.9%)	0.27
	TAC	13 (68.4%)	12 (57.1%)	0.46
	DFZ	10 (52.6%)	2 (9.5%)	0.003
	Pred	8 (42.1%)	13 (61.9%)	0.21
	Aza	5 (26.3%)	2 (9.5%)	0.16
	MMF	4 (21.1%)	4 (19%)	0.87

*Note: CI* calcineurin inhibitor, *ST* steroid, *AP* antiproliferative drug, *CyA* cyclosporine, *TAC* tacrolimus, *DFZ* deflazacort, *Pred* predinosolone, *Aza* azathioprine, *MMF* mycophenolate mofetil

**Table 3:** Differences in immunosuppressant use and the method of conception

		ART (*n* = 6)	Natural conception (*n* = 15)	*P* value
				0.08
Regimen	CI	1 (16.7%)	5 (33.3%)	
	CI + ST + AP	3 (50%)	2 (13.3%)	
	CI + ST	1 (16.7%)	8 (53.3%)	
	CI + AP	1 (16.7%)	0	
Immunosuppressant use	CyA	1 (16.7%)	8 (53.3%)	0.13
	TAC	5 (83.3%)	7 (46.7%)	0.13
	DFZ	2 (33.3%)	0	0.02
	Pred	3 (23.1%)	10 (66.7%)	0.48
	Aza	1 (16.7%)	1 (16.7%)	0.48
	MMF	3 (50%)	1 (6.7%)	0.02

*Note: ART* assisted reproductive techniques, *CI* calcineurin inhibitor, *ST* steroid, *AP* antiproliferative drug, *CyA* cyclosporine, *TAC* tacrolimus, *DFZ* deflazacort, *Pred* predinosolone, *Aza* azathioprine, *MMF* mycophenolate mofetil

Lastly, multivariate logistic models, using the Firth-type bias-reduced method, were utilized for some factors. The univariate analyses showed that the use of DFZ and whether the patient experienced graft rejection at the point of data collection were significantly different between the clinical pregnancy and non-pregnant groups (Table 4). After adjusting confounding factors, DFZ was the only significant immunosuppressant showing decreased odds to clinical pregnancy (odds ratio (OR) 0.06, 95% confidence interval (CI) 0.01–0.68, *P* = 0.02, Table 5). To examine the hazard factors for clinical pregnancy, Cox regression models were used (Table 6). Before adjustment, whether the patient experienced graft rejection or not at the point of data collection was a significant hazardous factor of clinical pregnancy. On the multivariate Cox model, DFZ use was the only significant hazardous factor for clinical pregnancy (hazard ratio (HR) 0.17, 95% CI 0.03–0.86, *P* = 0.03, Table 6). When the total duration and cumulative dosage of DFZ used were included in the respective models, the longer duration hindered clinical pregnancy (HR 0.97, 95% CI 0.95–0.99, *P* = 0.04, Table 6). For the cases that used DFZ, the total duration of DFZ use was 57 months (median; 25–188 months, min-max) and its cumulative dose was 12,051 mg (median; 672–68,940 mg, min-max).

**Table 4:** Unadjusted odds ratios of factors in clinical pregnancy

Factors	Unadjusted OR (95% CI)	*P* value
Age (years)	0.87 (0.72–1.06)	0.17
Duration tried to conceive (months)	0.98 (0.96–1.0)	0.09
Years since transplantation (years)	0.94 (0.80–1.10)	0.45
Hypertension	1.48 (0.37–5.96)	0.58
Serum BUN (mg/dL)	0.94 (0.87–1.03)	0.19
eGFR	1.03 (0.99–1.07)	0.09
Graft rejection	0.12 (0.02–0.65)	0.01
Diabetes mellitus	0.16 (0.004–7.09)	0.35
Cause of ESRD		
Unknown	1	
SLE	0.07 (0.002–2.49)	0.15
Glomerulonephritis	0.93 (0.16–5.38)	0.94
Ig A nephropathy	1.36 (0.2–9.56)	0.75
Congenital anomaly	0.13 (0.002–6.82)	0.31
Diabetes melitus	0.16 (0.001–25.91)	0.48
Use of ART	0.36 (0.1–1.33)	0.13
CyA	2.1 (0.55–8.0)	0.28
TAC	0.62 (0.17–2.25)	0.46
DFZ	0.09 (0.02–0.53)	0.007
Pred	2.23 (0.63–7.93)	0.21
Aza	0.29 (0.05–1.75)	0.18
MMF	0.88 (0.19–4.16)	0.88

*Note: ART* assisted reproductive techniques, *SBP* systolic blood pressure, *DBP* diastolic blood pressure, *BUN* blood urea nitrogen, *eGFR* estimated glomerular filtration rate, *ESRD* end stage renal disease, *SLE* systemic lupus erythematous, *Ig* immunoglobulin, *CyA* cyclosporine, *TAC* tacrolimus, *DFZ* deflazacort, *Pred* predinosolone, *Aza* azathioprine, *MMF* mycophenolate mofetil, *OR* odds ratio, *CI* confidence interval

**Table 5:** Adjusted odds ratios of immunosuppressants in clinical pregnancy

Immunosuppressant	Unadjusted OR (95% CI)	*P* value	Adjusted OR (95% CI)	*P* value
CyA	2.1 (0.55–8.0)	0.28	1.38 (0.22–8.89)	0.73
TAC	0.62 (0.17–2.25)	0.46	0.84 (0.16–4.49)	0.84
DFZ	0.09 (0.02–0.53)	0.007	0.06 (0.01–0.68)	0.02
Pred	2.23 (0.63–7.93)	0.21	2.8 (0.53–14.85)	0.23
Aza	0.29 (0.05–1.75)	0.18	0.48 (0.06–4.1)	0.5
MMF	0.88 (0.19–4.16)	0.88	0.78 (0.14–4.39)	0.78

The *P*-values were obtained using simple and multiple logistic regression analysis. The analysis was adjusted for age, estimated glomerular filtration rate, graft rejection after transplantation, diabetes mellitus, and use of assisted reproductive techniques. The logistic regression analysis was performed for each immunosuppressant, respectively

*Note*: CyA, cyclosporine; TAC, tacrolimus; DFZ, deflazacort; Pred, predinosolone; Aza, azathioprine; MMF, mycophenolate mofetil; OR, odds ratio; CI, confidence interval

**Table 6:** Adjusted hazard ratios of immunosuppressants for clinical pregnancy and duration

Immunosuppressant	Unadjusted HR (95% CI)	*P* value	Adjusted HR (95% CI)	*P* value
CyA	0.89 (0.37–2.14)	0.8	0.33 (0.1–1.09)	0.07
TAC	1.16 (0.49–2.79)	0.73	2.99 (0.92–9.72)	0.07
DFZ	0.23 (0.05–0.99)	0.05	0.17 (0.03–0.86)	0.03
Pred	1.28 (0.53–3.11)	0.59	1.06 (0.42–2.72)	0.9
Aza	0.31 (0.07–1.34)	0.12	0.36 (0.08–1.77)	0.21
MMF	0.57 (0.19–1.72)	0.32	0.61 (0.2–1.83)	0.38
Cumulative dose of DFZ (mg)	1.0	0.11	1.0	0.14
Total duration of DFZ use (months)	0.98 (0.95–1.0)	0.06	0.97 (0.95–1.0)	0.04

The *P*-values were obtained using univariate and multivariate cox regression analyses. The analysis was adjusted for age, eGFR, graft rejection after transplantation, diabetes mellitus, and use of assisted reproductive techniques. The cox regression multivariate analysis was performed for each immunosuppressant, respectively

*Note*: *CyA* cyclosporine, *TAC* tacrolimus, *DFZ* deflazacort, *Pred* predinosolone, *Aza* azathioprine, *MMF* mycophenolate mofetil, *HR* hazard ratio, *CI* confidence interval

## Discussion

Considering the lack of knowledge in how immunosuppressants affect successful pregnancy, our study suggests a possible detrimental effect of DFZ for the first time. Until now, most studies on the adverse effects of immunosuppressants have focused on the development of diabetes or osteoporosis with their long-term use. Some studies have shown their impact on pregnancy complications and fetal outcome; but studies studying their impact on either fertility or pregnancy success are few. Our study suggests that the type of immunosuppressant regimen may not affect the success of clinical pregnancy; however, a certain medication-DFZ- may.

In our study, of the 40 cases, 17 cases were on CI and ST regimen (17/40, 42.5%), 12 on CI with ST and AP regimen (12/40, 30%), 9 on CI regimen only (9/40, 22.5%), and 2 on CI and AP regimens (2/40, 5%). Little is known on the effect of immunosuppressants on fertility. AZT was reported to show teratotoxicity in animal studies, but not in humans [12]. MMF is a reversible inhibitor of inosine monophosphate dehydrogenase, which blocks de novo purine synthesis and is thereby suggested as a category D drug in pregnancy. An increased risk of miscarriage and congenital orofacial malformations, especially microtia, is reported with MMF use [7]. CI acts by blocking cytokine secretion necessary for T-cell activation and proliferation. CyA and TAC are category C drugs in pregnancy that require strictly monitored serum levels. There has been no evidence of congenital anomaly development with these drugs; however, alteration of the immune response in neonates exposed to TAC in utero is possible [13]. Corticosteroids are mostly category B drugs in pregnancy that are associated with multiple maternal adverse effects [14]. Although the effect of Pred use is still unclear, it has been suggested that it enhances implantation rate in infertile women and decreases the risk of miscarriage in women with idiopathic recurrent pregnancy loss [15, 16]. The effect of Pred on uterine natural killer (NK) cells may be the key factor to its positive effect. Pred may inhibit uterine NK cells in the endometrium [16] and bind to the glucocorticoid receptor in the endometrium, which leads to an immunomodulating effect [17].

DFZ is a heterocyclic corticosteroid, which is an oxazoline derivative of Pred. It was developed in the early 1980s [18] to treat patients with Duchenne muscular dystrophy [19] and is characterized by high efficacy and good tolerability because of its substantial lack of fluid retention and low interference with carbohydrate and phosphocalcium metabolisms [20]. Given DFZ’s excellent anti-inflammatory properties and good tolerability, it is preferred in patients with osteoporosis [21] or diabetes [22]. In our study, there were 2 patients with diabetes among those 12 patients who used DFZ. Although the number of patients using immunosuppressants is consistently increasing, little is known of their long-term effects during pregnancy. This may be because the indication for DFZ use was initially for patients with Duchenne muscular dystrophy who are less likely to attempt conception because of their underlying disease. As DFZ use is increasing in various fields of autoimmune diseases [23, 24], comparison to Pred has been done in terms of osteoporosis, cushinoid features, and body weight [25]. Our study is the first and only study, to our knowledge, showing adverse effects regarding fertility in those who use DFZ.

Since the purpose of the study was to identify whether there is any need to change the immunosuppressant before trying to conceive, we generated logistic and Cox hazard models that focused on each immunosuppressant. To minimize the interaction among drugs and to focus on the effect of each immunosuppressant, multivariate models were constructed to include each immunosuppressant separately with the factors that showed significance on univariate analyses. DFZ showed significantly decreased odds for clinical pregnancy (OR 0.06, *P* = 0.02) and decreased possibility for achieving clinical pregnancy (HR 0.17, *P* = 0.03) after adjustment on multivariate models. Considering studies which suggest the positive effects of steroids on pregnancy, our result showing the possible detrimental effect of DFZ is unexpected. However, the possible harm of DFZ on conception may be explained by the difference between Pred and DFZ in inhibiting type 1 11ß-hydroxysteroid dehydrogenase (type 1 11ß-HSD) [26]. Type 1 11ß-HSD is an enzyme widely distributed throughout the central nervous system and peripheral tissues, and it is essential in hepatic and adipose tissue carbohydrate metabolism [27]. Decreased type 1 11ß-HSD expression was significantly associated with increased uterine NK cell density especially in the human endometrium [28]. Moreover, type 1 11ß-HSD has been identified as a key factor in decidualization of endometrial stromal cells [29]. The difference in the inhibiting ability of type 1 11ß-HSD may lead to a different expression of uterine NK cells and may affect the process of decidualization in the endometrium, resulting in a detrimental effect in implantation and successful pregnancy.

In the current study, renal function was better in the clinical pregnancy group than in the miscarriage group, exhibiting a significantly high eGFR and low serum BUN level (Table 1). However, the factors which may reflect graft function, such as eGFR and graft rejection, were not the ones with affected odds or hazard after adjustment for clinical pregnancy. Better kidney function with fewer graft rejection should be associated with better pregnancy prognosis, resulting in a lower possibility of miscarriage; however, our study did not prove this hypothesis. After discovering the possible detrimental effect of DFZ on clinical pregnancy, we additionally analyzed whether the duration or cumulative dosage of DFZ may further affect the clinical pregnancy. The total duration of DFZ use showed a possible hazardous impact (Table 6); however, the hazard was not found to be significant, probably because of the small number of cases included in this study. There were no cases of newly developed acute rejection during the period of trying to conceive.

Our study was subject to several limitations. First, our study had a small number of patients and was of retrospective design. There may be a selective bias as kidney transplant patients who failed to conceive, but did not seek fertility treatment might be missed. However, as kidney transplant patients are usually very concerned about how their fertility is effected by their medication use, we believe that most would discuss infertility at length with their primary physician and seek further help if needed. Nonetheless, it may be unclear as to whether infertility in these patients is due to the immunosuppressant use or the fact that they did not seek fertility treatment. Secondly, as DFZ use was limited to certain diseases and countries, long-term reference data was unavailable. Finally, it is unclear as to how the immunosuppressants may interact with each other and whether they will negatively impact fertility. On the other hand, the strength of our study is that, for the first time, we showed a possible detrimental effect of DFZ in terms of implantation and success of pregnancy. To our knowledge, excluding the report demonstrating TAC as a basic immunosuppressive agent that achieved a high rate of successful pregnancy [30], this is the only study that investigated the effect of immunosuppressant use in fertility and pregnancy outcome.

## Conclusions

In conclusion, DFZ may decrease the possibility of clinical pregnancy. The use and effect of immunosuppressants need to be evaluated more thoroughly in future studies. Before attempting pregnancy, modulating the immunosuppressant regimen may be required.

## Abbreviations

AMH: Anti-Müllerian hormone
AP: antiproliferative drug
AZT: azathioprine
BP: blood pressure
BUN: blood urea nitrogen
CI: calcineurin Inhibitor
Cr: creatinine
CyA: cyclosporine
DFZ: deflazacort
ESRD: end-stage renal disease
IUI: intrauterine insemination
IVF: in vitro fertilization
MMF: mycophenolate mofetil
NK: natural killer
Pred: prednisolone
SLE: systemic lupus erythematous
TAC: tacrolimus
TVS: transvaginal ultrasonography
type 1 11ß-HSD: type 1 11ß-hydroxysteroid dehydrogenase

## References

[1] Murray JE, Reid DE, Harrison JH, Merrill JP (1963). Successful pregnancies after human renal transplantation. N Engl J Med.

[2] Lockwood GM, Ledger WL, Barlow DH (1995). Successful pregnancy outcome in a renal transplant patient following in-vitro fertilization. Hum Reprod.

[3] Shah S, Verma P (2016). Overview of pregnancy in renal transplant patients. International journal of nephrology.

[4] Lopez LF, Martinez CJ, Castaneda DA, Hernandez AC, Perez HC, Lozano E (2014). Pregnancy and kidney transplantation, triple hazard? Current concepts and algorithm for approach of preconception and perinatal care of the patient with kidney transplantation. Transplant Proc.

[5] Lessan-Pezeshki M, Ghazizadeh S, Khatami MR, Mahdavi M, Razeghi E, Seifi S, Ahmadi F, Maziar S (2004). Fertility and contraceptive issues after kidney transplantation in women. Transplant Proc.

[6] Cardonick E, Moritz M, Armenti V (2004). Pregnancy in patients with organ transplantation: a review. Obstet Gynecol Surv.

[7] Mastrobattista JM, Gomez-Lobo V, Society for Maternal-Fetal M (2008). Pregnancy after solid organ transplantation. Obstet Gynecol.

[8] Maesawa Y, Yamada H, Deguchi M, Ebina Y (2015). History of biochemical pregnancy was associated with the subsequent reproductive failure among women with recurrent spontaneous abortion. Gynecol Endocrinol.

[9] Sienko J, Jasiczek A, Paczek L, Wyczalkowska-Tomasik A, Kotowski M, Nowacki A, Sulikowski T, Romanowski M, Ostrowski M (2014). Evaluation of renal graft function based on standard mathematical formulas. Ann Transplant.

[10] Abouchacra S, Chaaban A, Hakim R, Gebran N, El-Jack H, Rashid F, Boobes Y, Muhairi A, Hussain Q, Khan I (2012). Renal biomarkers for assessment of kidney function in renal transplant recipients: how do they compare?. Int Urol Nephrol.

[11] Tocci A, Lucchini C (2010). WHO reference values for human semen. Hum Reprod Update.

[12] Armenti VT, Radomski JS, Moritz MJ, Gaughan WJ, Gulati R, McGrory CH, Coscia LA. Report from the National Transplantation Pregnancy Registry (NTPR): outcomes of pregnancy after transplantation. Clin Transpl. 2005:69–83. 17424726

[13] Mak RH, Hoffman HM (2015). Transplantation: outcomes of prenatal immunosuppression. Nat Rev Nephrol.

[14] Tedeschi SK, Guan H, Fine A, Costenbader KH, Bermas B (2016). Organ-specific systemic lupus erythematosus activity during pregnancy is associated with adverse pregnancy outcomes. Clin Rheumatol.

[15] Dan S, Wei W, Yichao S, Hongbo C, Shenmin Y, Jiaxiong W, Hong L (2015). Effect of prednisolone administration on patients with unexplained recurrent miscarriage and in routine intracytoplasmic sperm injection: a meta-analysis. Am J Reprod Immunol.

[16] Kemp MW, Newnham JP, Challis JG, Jobe AH, Stock SJ (2016). The clinical use of corticosteroids in pregnancy. Hum Reprod Update.

[17] Henderson TA, Saunders PT, Moffett-King A, Groome NP, Critchley HO (2003). Steroid receptor expression in uterine natural killer cells. J Clin Endocrinol Metab.

[18] Hahn BH, Pletscher LS, Muniain M (1981). Immunosuppressive effects of deflazacort - a new glucocorticoid with bone-sparing and carbohydrate-sparing properties: comparison with prednisone. J Rheumatol.

[19] Matthews E, Brassington R, Kuntzer T, Jichi F, Manzur AY (2016). Corticosteroids for the treatment of Duchenne muscular dystrophy. Cochrane Database Syst Rev.

[20] Scudeletti M, Castagnetta L, Imbimbo B, Puppo F, Pierri I, Indiveri F (1990). New glucocorticoids. Mechanisms of immunological activity at the cellular level and in the clinical setting. Ann N Y Acad Sci.

[21] Kim MS, Kim YS, Lim SK, Kim SI, Moon JI, Park K (1998). Effect of deflazacort on bone mineral density in renal transplant recipients. Transplant Proc.

[22] Kim YS, Kim MS, Kim SI, Lim SK, Lee HY, Han DS, Park K (1997). Post-transplantation diabetes is better controlled after conversion from prednisone to deflazacort: a prospective trial in renal transplants. Transpl Int.

[23] Bakthavatchalam M, Lai FHP, Rong SS, Ng DS, Brelen ME (2018). Treatment of cystoid macular edema secondary to retinitis pigmentosa: a systematic review. Surv Ophthalmol.

[24] Shieh PB, McIntosh J, Jin F, Souza M, Elfring G, Narayanan S, Trifillis P, Peltz SW, McDonald CM, Darras BT (2018). Deflazacort versus prednisone/prednisolone for maintaining motor function and delaying loss of ambulation: a post HOC analysis from the ACT DMD trial. Muscle Nerve.

[25] Ganapati A, Ravindran R, David T, Yadav B, Jeyaseelan V, Jeyaseelan L, Danda D. Head to head comparison of adverse effects and efficacy between high dose deflazacort and high dose prednisolone in systemic lupus erythematosus: a prospective cohort study. Lupus. 2018:961203317751854. 10.1177/0961203317751854 29320974

[26] Hult M, Jörnvall H, Oppermann UC (1998). Selective inhibition of human type 1 11beta-hydroxysteroid dehydrogenase by synthetic steroids and xenobiotics. FEBS Lett.

[27] Voice MW, Seckl JR, Edwards CR, Chapman KE (1996). 11 beta-hydroxysteroid dehydrogenase type 1 expression in 2S FAZA hepatoma cells is hormonally regulated: a model system for the study of hepatic glucocorticoid metabolism. Biochem J.

[28] Kuroda K, Venkatakrishnan R, James S, Sucurovic S, Mulac-Jericevic B, Lucas ES, Takeda S, Shmygol A, Brosens JJ, Quenby S (2013). Elevated periimplantation uterine natural killer cell density in human endometrium is associated with impaired corticosteroid signaling in decidualizing stromal cells. J Clin Endocrinol Metab.

[29] Kuroda K, Venkatakrishnan R, Salker MS, Lucas ES, Shaheen F, Kuroda M, Blanks A, Christian M, Quenby S, Brosens JJ (2013). Induction of 11beta-HSD 1 and activation of distinct mineralocorticoid receptor- and glucocorticoid receptor-dependent gene networks in decidualizing human endometrial stromal cells. Mol Endocrinol.

[30] Garcia-Donaire JA, Acevedo M, Gutierrez MJ, Manzanera MJ, Oliva E, Gutierrez E, Andres A, Morales JM (2005). Tacrolimus as basic immunosuppression in pregnancy after renal transplantation. A single-center experience. Transplant Proc.

